# Chitosan and Pectin Hydrogels for Tissue Engineering and In Vitro Modeling

**DOI:** 10.3390/gels9020132

**Published:** 2023-02-04

**Authors:** Giulia Morello, Gianvito De Iaco, Giuseppe Gigli, Alessandro Polini, Francesca Gervaso

**Affiliations:** 1 Dipartimento di Matematica e Fisica E. De Giorgi, University of Salento, c/o Campus Ecotekne, Via Monteroni, 73100 Lecce, Italy; 2 CNR NANOTEC—Institute of Nanotechnology, c/o Campus Ecotekne, Via Monteroni, 73100 Lecce, Italy

**Keywords:** natural polymer, chitosan and pectin biopolymers, thermoresponsive hydrogel, semi-IPN network, 3D in vitro models, tissue engineering

## Abstract

Hydrogels are fascinating biomaterials that can act as a support for cells, i.e., a scaffold, in which they can organize themselves spatially in a similar way to what occurs in vivo. Hydrogel use is therefore essential for the development of 3D systems and allows to recreate the cellular microenvironment in physiological and pathological conditions. This makes them ideal candidates for biological tissue analogues for application in the field of both tissue engineering and 3D in vitro models, as they have the ability to closely mimic the extracellular matrix (ECM) of a specific organ or tissue. Polysaccharide-based hydrogels, because of their remarkable biocompatibility related to their polymeric constituents, have the ability to interact beneficially with the cellular components. Although the growing interest in the use of polysaccharide-based hydrogels in the biomedical field is evidenced by a conspicuous number of reviews on the topic, none of them have focused on the combined use of two important polysaccharides, chitosan and pectin. Therefore, the present review will discuss the biomedical applications of polysaccharide-based hydrogels containing the two aforementioned natural polymers, chitosan and pectin, in the fields of tissue engineering and 3D in vitro modeling.

## 1. Introduction

In recent years, the world of scientific research has witnessed the development of 3D tissue analogues which are not only excellent tools for restoring diseased biological tissues and organs, but also for studying the physiology and diseases of a specific organ or tissue in vitro as an alternative to classical animal models and 2D in vitro cultures. When a biological tissue suffers injury because of trauma or pathologies, our body tries to self-repair the defect, but this process is not always successful. To fully understand the mechanisms behind tissue regeneration or pathological events, and also to test new therapeutics to promote regeneration and healing, it is of paramount importance to rebuild tissues in vitro by obtaining tissue analogues. Though cells are undoubtedly an essential component of a tissue, a 3D structure, mimicking the extracellular matrix (ECM) architecture, is crucial to recreate the spatial organization of the original tissue, supporting cell migration, proliferation, and differentiation. Hydrogel systems, defined as three-dimensional, hydrophilic, polymeric networks capable of absorbing large amounts of water or biological fluids [1] of up to thousands of times of their dry weight [2], can serve as 3D structures devoted to host cells, since they are able to recapitulate the dynamic nature of the ECM, similar to what occurs in vivo. By carefully selecting the hydrogel material components, it is possible to tune hydrogel porosity, degradation, and mechanical and surface properties to meet the requirements of the tissue to rebuild and make the cells feel at home. 

According to their composition, hydrogel systems can be classified into natural, including polysaccharides, proteins, and animal derivatives (e.g., alginate, chitosan, hyaluronic acid, pectin, and collagen), synthetic (e.g., polyethylene glycol (PEG), polylactic acid (PLA), poly(lactic-co-glycolic acid) (PLGA), and polycaprolactone (PCL)), or hybrid matrices (i.e., a combination of thereof) [3,4]. Due to their bioinert properties, synthetic hydrogels are biomaterials widely applied to cell cultures and, unlike natural polymers, they display excellent biochemical properties, stiffness, and density [5,6,7,8,9]. However, because they are biologically inactive materials, they require functionalization processes with ECM proteins or amino acid sequences to increase their biocompatibility and enable matrix remodeling [10,11,12]. Furthermore, these matrices represent static substrates that do not simulate the interactions present in ECM well [13]. In contrast to synthetic systems, natural hydrogels can establish a better interaction with living cells, in view of their composition, similar to the ECM of a specific organ or tissue [14]. Due to their biodegradability, their ability to gel under non-extreme conditions, and their physicochemical properties, animal-derived mixtures are often used as matrices for 3D cell cultures. Among animal ECM materials, the most widely used in 3D cultures are Matrigel and its derivates [15]. Matrigel is a gelatinous protein mixture derived from extracts of Engelbreth-Holm-Swarm mouse tumors. It is an excellent biomaterial, commonly used in organoid cultures because of its physiological characteristics and nature [7]. However, the poor stability and high biodegradability of these matrices generally make them unsuitable for long-term culture [16]. To overcome Matrigel limitations, other natural polymers, such as polysaccharides, have been investigated and tested as 3D tissue analogues [17,18,19,20]. Polysaccharide hydrogels are characterized by excellent biocompatibility and biodegradability, aspects of primary importance for tissue engineering applications and 3D in vitro modeling [21]. Among the polysaccharides, pectin and chitosan have recently gained an increasing interest due to their marked nontoxic nature and their biodegradable nature. Although the use of polysaccharide-based hydrogels in the biomedical field is evidenced by a substantial number of reviews on the topic [22,23,24,25,26,27], none of them have focused on the combined use of these two important polysaccharides. In the light of the above considerations, and with the aim of shedding light on the promising combined use of chitosan and pectin, the present review will focus on the biomedical applications of polysaccharide-based hydrogels containing the two aforementioned natural polymers, chitosan and pectin, in the field of tissue engineering and 3D in vitro modeling.

**Figure 1:**
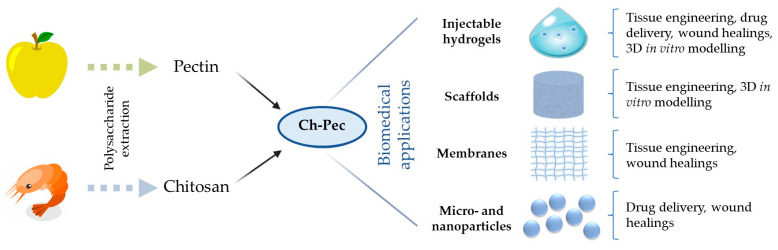
Scheme illustrating chitosan and pectin source of extraction and their combined used for biomedical applications.

In more detail, the present review will provide a broad perspective on hydrogels as injectable scaffolds for biological tissue regeneration and 3D platforms to model diseases in vitro. Moreover, polysaccharide hydrogels, focusing on chitosan, pectin, and their composites, will be introduced and their promising use in the field of tissue engineering and 3D culture systems will be finally highlighted. Since the use of chitosan- and pectin-based hydrogels (pure or combined with natural/synthetic polymers) has been widely investigated for biomedical applications and exhaustively reviewed [22,23,24,25,26,27], the present review will focus on chitosan/pectin hybrid systems only (Figure 1).

## 2. Hydrogels as 3D Platforms for Tissue Engineering and In Vitro Model Applications

In the past decades, tissue engineering has emerged with the aim of repairing and improving the function of a damaged organ or tissue [28]. Of fundamental importance in the tissue regeneration processes is to provide cells with a 3D environment, i.e., ECM analogues, that can promote cell growth and maintain cell metabolism by allowing the exchange of nutrients, gases, and waste metabolites [29,30].

To this purpose, hydrogel systems are widely used in tissue engineering since, due to some of their specific properties such as viscoelasticity and oxygen and metabolite permeability, they structurally resemble the extracellular matrix of biological tissues [31,32]. Indeed, hydrogels exhibit certain characteristics that are fundamental requirements for tissue engineering applications, including biodegradability during tissue formation and excellent porosity, as well as being nontoxic and biocompatible. Last but not least, it is necessary that these biomaterials ensure adhesion, gene expression, and cell proliferation [33]. 

Biopolymer-based hydrogels are widely used in the tissue engineering field because they closely resemble the extracellular matrix of a tissue [34], but also hybrid hydrogels, composed of two or more polymeric components, have been considered good candidates for tissue reconstruction in tissue engineering, since they showed the promotion of cell attachment, growth, and proliferation [35].

Hydrogels have been extensively used both in vitro and in vivo to form scaffolds in which cells can self-organize, proliferate, rebuild the target tissue ECM, and promote the regeneration of various types of tissues, including liver, neurons and muscles, as well as cartilage and bone [36]. They have also been widely employed as delivery systems supporting the transport of drugs or bioactive molecules involved in tissue repair processes [37]. Regarding the applications of hydrogels in vivo, injectable systems are extremely interesting and have been explored for several applications. The main advantage of injectable hydrogels is that being in the liquid state before injection, they can be easily inserted into the damaged tissue/defect mini-invasively through a syringe needle, hence reaching the “solid” state by the effect, for example, of the physiological temperature or the time. Compared with traditional structured scaffolds, injectable hydrogels are less invasive and at the same time can be exploited to deliver biomolecules and soluble factors that can boost the damaged tissue repair [38]. Injectable hydrogels coupled with controlled drug release systems have been proposed for ophthalmological applications, for wound and burn healing, and also for cartilage and bone regeneration [39]. Injectable hydrogels are indeed promising candidates in reconstructive surgery and drug delivery, providing a suitable 3D matrix to host cell ingrowth and neovascularization. However, the clinical approval of currently developed injectable systems remains low, often due to the strict preparation requirements, device malfunction, product dislodgment during administration, and uncontrolled biological responses at the treatment site [40]. Fully synthetic and ready-to-use injectable biomaterials can help in overcoming some of the above-highlighted issues toward the clinical translation of hydrogels, but they overall show undoubtedly lower cell affinity.

In addition to their extensive use in the field of tissue engineering, in recent years hydrogels have seen a large application as 3D culture systems in vitro. The use of in vitro models is essential to study the biological process involved in the physiopathology of tissues and organs and, in this context, 3D culture systems can represent an excellent platform for studying unknown biological phenomena, disease onset and evolution, and testing new drugs and therapies, providing a promising alternative to traditional two-dimensional cultures and animal models. In the progression of a disease, a key role is played by the complex mechanisms of interaction that take place between the cellular components and the microenvironment, which present specific characteristics in a given tissue [41]. 3D matrices, such as hydrogels, allow to recapitulate the cell microenvironment providing the structural support needed by cells to organize themselves in a similar way to what is observed in vivo, thus developing a more realistic and physiologically relevant model [42]. 

Due to their versatility, polymer-based hydrogels are a promising tool for the in vitro study of disease mechanisms. The huge advances in materials engineering have provided the field of 3D modeling with polymer biomaterials that can promote cell aggregation in three-dimensional clusters and support cell adhesion and growth due to their defined biochemical and mechanical properties. In this regard, several studies have proposed hydrogels as platforms for studying complex physiological and pathological conditions that cannot be faithfully replicated by 2D in vitro cultures, such as cancer [43,44] or neurodegenerative diseases [45,46]. Different strategies can be adopted to generate 3D cell/hydrogel systems; for example, spheroids can be previously formed by conventional techniques (such as hanging drop or by using ultra-low attachment well plates) and then embedded within the polymer network [47], or single cells can be directly enclosed in the polymer solution and spheroids spontaneously generate inside the hydrogel network [48].

Moreover, in recent years, 3D cultures have also been combined in high-throughput drug screening, with the aim of better predicting drug efficacy and improving the selection of the best performing candidate drugs to be used in subsequent in vivo testing on animals [49,50]. 

Furthermore, a growing interest has recently arisen in the study and comprehension of the impact of local mechanical environment on cell response, that is known to be strongly influenced by ECM mechanical properties [51]. Hydrogels that mimic the native ECM represent indeed a suitable 3D environment in this direction, being able to provide different mechanical cues and allow mechanobiology studies. Moreover, once the relationship between cellular responses and mechanical cues is assessed, hydrogels can be successfully used to create tissues in vitro for regenerative medicine applications and for disease modeling [52]. In fact, in the context of tissue engineering applications, the major goal is to design hydrogels with biological and mechanical properties that closely resemble those of the native ECM to be reconstructed. One of the peculiar mechanical characteristics of a hydrogel is its viscoelastic behavior, a feature that most of the biological tissues also show. It has been shown that hydrogels exhibiting time-dependent mechanical behaviors significantly enhance cell migration, proliferation, and differentiation compared to elastic hydrogels. In the overall evaluation of hydrogel physico-chemical properties, an accurate determination of their time-dependent behaviors is therefore crucial. Rheology is the most used technique for this [34], although other techniques, such as AFM, have been proposed to probe the mechanical response at the cell level [53]. 

## 3. Polysaccharide-Based Hydrogels: Chitosan and Pectin

As previously highlighted, hydrogels composed of natural polymers have found a wide application in the field of tissue engineering and 3D in vitro modeling because of their great affinity to the extracellular matrix of biological tissues. Indeed, during the natural degradation process they do not release sub-products that can be toxic to cells, showing high biocompatibility and bioactivity [54]. Moreover, natural polymer-based biomaterials have shown to support different cellular processes and ensure biological recognition [54]. Among the plethora of natural polymers, this review focuses on pectin and chitosan; they are particularly interesting as they are biodegradable, non-toxic, largely available, low cost and, as a result of all these features, find wide use in tissue engineering and 3D cell culture. 

### 3.1. Chitosan

Chitosan (Ch) is a naturally occurring polymer derived from a highly bioavailable mucopolysaccharide called chitin, present in the exoskeleton of some shellfish (shrimps, lobsters, and crabs) and in some mycelia [55,56,57,58,59,60,61]. Ch is the N-deacetylated form of chitin [56,58,59,61], and similar to chitin itself, it is biocompatible, non-toxic, basic, biodegradable, and displays excellent absorption [58,59], antibacterial, and antifungal properties [59].

**Figure 2:**
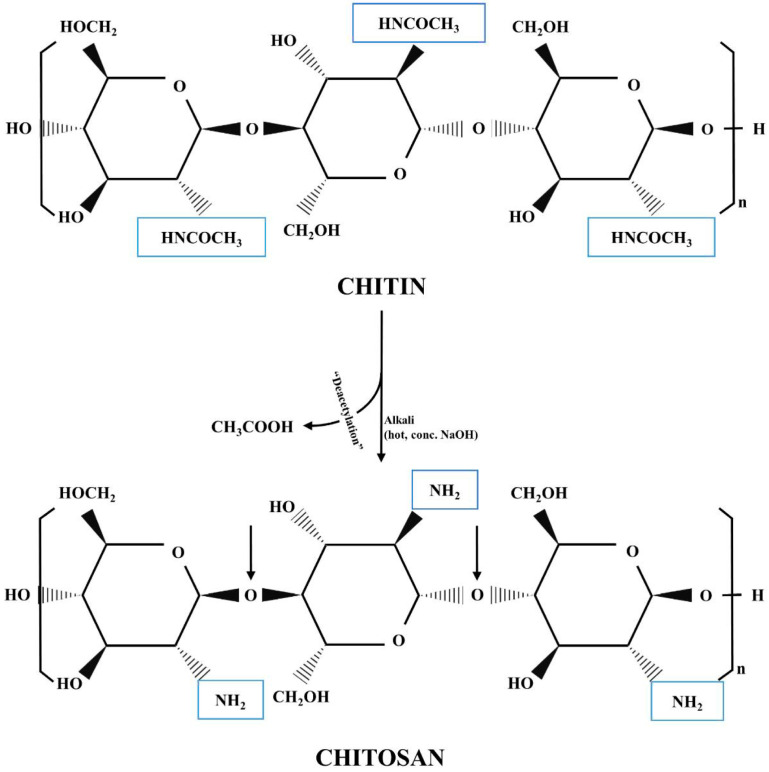
Comparison of the chemical structure of chitin and chitosan [62].

Chitin behaves as a structural cationic and linear polysaccharide and consists of the succession of 2-acetamido-2-deoxy-β-D-glucose units, held together by β (1-4) glycosidic bonds (Figure 2) [58,59]. It is characterized by a white, hard, inelastic appearance and performs similar to cellulose, both in terms of low solubility and low reactivity from the chemical point of view [58]. Chitin is obtained from the shells of crustaceans, which is then deacetylated for 1–3 h in 40% sodium hydroxide (NaOH) at high temperatures (120 °C) to obtain, finally, Ch. In addition, 70% deacetylated Ch can be obtained through the removal of protein and calcium carbonate [58].

Due to its biodegradability and biocompatibility, Ch is a biomaterial that finds application in different biomedical fields, and its wound-healing, anti-microbial, and anti-tumor properties make it an excellent candidate for the development of injectable pharmaceuticals and scaffolds for tissue regeneration [63,64,65,66,67,68,69,70]. Unlike chitin, which is insoluble in water and most organic solvents, Ch is soluble in dilute acid solutions [58]. Indeed, the solubilization of Ch occurs only in an acidic environment and, more specifically, the use of strong bases allows the neutralization of its positive charges, leading to its precipitation. On the contrary, it is possible to obtain a deacetylated Ch in solution at physiological pH using a weak base. This allows the polymer to gel and form a thermoreversible gel [71].

Ch exhibits an excellent ability to gel in the presence of weak bases at physiological temperature through interactions that are created between its amine groups and the salts used. Weak bases include β-glycerophosphate (βGP), sodium bicarbonate (NaHCO_3_), sodium dibasic phosphate (SPD), and sodium hydrogen carbonate (SHC) [55]. In detail, βGP exhibits a pKa (acid dissociation constant) of 6.65 at 25 °C, similar to the pKa of Ch (6.5); this allows Ch to remain soluble at a pH around neutrality at room temperature and to transform in the presence of salt into an injectable hydrogel by increasing the temperature. The mechanism of gelation is induced by the fact that heat triggers the neutralization of Ch, with the transfer of protons from Ch to glycerolphosphate. As a result, the interaction between Ch chains increases, since the repulsive forces between positively charged amine groups decrease [72].

In addition, the degree of acetylation, as well as the type and concentration of gelling agent (βGP) and polymer, exert an influence on the rheological and physicochemical properties and on the gelling process, which is related to the increase in temperature to 37 °C [73]. Hydrogels created by chemical cross-linking of Ch with βGP have been used as platforms for chemotherapeutic drug delivery [74], as well as for the controlled release of nanoparticles loaded with anticancer drugs [75]. In contrast to classical Ch-βGP hydrogels, which present problems with cytotoxicity and gelation time, in a 2015 study by Assad and coworkers, a synergistic combination of multiple gelling agents allowed to obtain thermoresponsive and injectable Ch hydrogels by rapid gelation. These systems showed improved mechanical properties at a physiological pH and outstanding biocompatibility [55]. The ability of Ch to interact with different gelling agents, i.e., βGP and SHC, has been exploited to obtain thermosensitive injectable hydrogels for the development of long-term 3D in vitro disease models [76]. Similar hydrogels have been further optimized to avoid any bubble entrapment during the preparation of cell-laden formulation by a drop-by-drop method. The resulting systems proved to be excellent candidates for creating in vitro 3D models of motor neuron diseases, allowing motor neuron-like cells to grow and differentiate inside the hydrogel [46].

Ch scaffolds have also been developed for performing 3D cell culture studies based on 3D cell aggregates (spheroids). In a study from 2006, a micro-molding approach for a photocrosslinkable Ch hydrogel, able to support spheroid formation from co-culture of hepatoblastoma (Hep G2) and fibroblast cells (NIH-3T3), was presented [77]. In another study, a Ch-based membrane modified with RGD sequences supported the formation of rat adipose-derived adult stem cell (ASC) spheroids, triggered to differentiate into cardiomyocytes and participate in tissue regeneration after injury [78]. Recently, an injectable and thermoresponsive collagen–Ch-based hydrogel was tested for wound healing in diabetic animals. After the formation of mesenchymal stem cell (MSCs), spheroids were encapsulated for a week in this hydrogel composition and were inoculated in vivo. This matrix was biocompatible, displayed good mechanical properties, and showed an optimal effect on proliferation and secretion of paracrine factors, which promoted in situ angiogenesis and wound re-epithelialization [79]. Moving towards 3D tumor models, Ch was shown to act as an intercellular linker to accelerate the development of multicellular liver cancer spheroids in a PEG-isopropylacrylamide-based thermoresponsive hydrogel [80]. A 3D culture matrix based on PEG-Ch with genipin, a natural cross-linking agent for naturally derived polymers, enabled the development of spheroids from glioblastoma cells within 2–3 days of culture; the system was better able to mimic brain tumor structure when compared to 2D systems and Matrigel [81].

Despite the presence of very interesting features, such as excellent biocompatibility and biodegradability, Ch shows quite low cell adhesion properties. In order to improve this limitation, Ch can be derivatized and several modification strategies have been proposed involving either the amino group (–NH_2_) or hydroxyl groups (–OH) [82]. Different Ch modifications have been explored to improve the cell–biomaterial interaction, by introducing short amino acid sequences present in the proteins of the ECM, such as RGD (arginine-glycin-aspartic acid), in the biopolymer, known to induce adhesion and migration through interaction with the integrin family. Functionalization of Ch with RGD peptide sequence was shown to increase the spread of human dermal fibroblasts in vitro and the interaction between the biomaterial and the surrounding tissue [83], leading to a significant improvement in adhesion (150%) and proliferation (300%) of human mesenchymal stem cells [84]. Moreover, lactose-modified Ch has recently emerged as a fascinating example of a biopolymer for potential applications in the field of tissue engineering [85,86]. Besides its interesting physico-chemical properties, lactose-modified Ch showed improved biological properties toward different types of primary cells, promoting chondrocytes aggregation [87], neuronal growth, differentiation, maturation, and formation of synapses, thus representing a good candidate for central nervous system regeneration [88]. 

### 3.2. Pectin

Pectin (Pec) is a linear, non-toxic, anionic plant polysaccharide characterized by the presence of galacturonic acid molecules that is widely used for biomedical applications [89,90]. This anionic polysaccharide has excellent gelling properties, is derived from natural resources as a structural constituent of the plant cell wall [91,92], and is characterized by a mucoadhesive and biocompatible nature [56,93].

**Figure 3:**
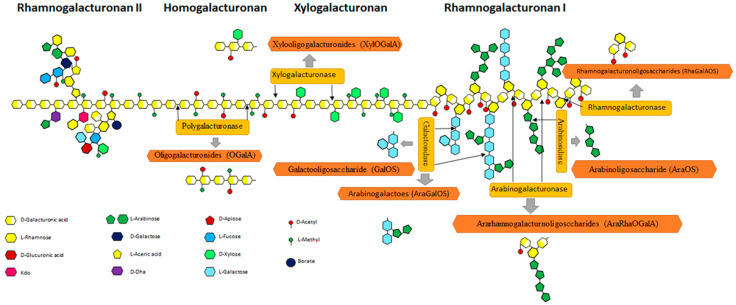
Representation of the chemical structure of Pec [96].

Since it is extremely complex, the chemical structure of Pec has not yet been fully determined [94]. Pec consists of a linear framework of α-D-galacturonic acid (Gal A) units, held together by α (1-4) glycosyl bonds [56,60,61]. Some of these units may exhibit methyl esterifications of carboxylic acid residues, through α-L-rhamnose residues joined by α (1-2) glycosidic bonds [61,93]. From a polymeric point of view, pectic substances can be classified into different forms such as homogalacturonan (HG), rhamnogalacturonan-I (RG-I), xylogalacturonan (XGA), and rhamnogalacturonan-II (RG-II), as shown in (Figure 3). HG makes up about 65% of Pec in plants and consists of linear chains of Gal A, some of which may be acetylated or methylated. Less than 10% of Pec in plants is represented by XGA, consisting of xylose side residues linked with β-1,4 covalent bonds. RG-I, on the other hand, constitutes about 20–35% of the Pec in plants, and appears to consist of a linear chain of Gal A branched rhamnose (Rha) residues, bound with α-1,2, glycosidic bonds and, in some cases, with lateral residues of galactan, branched arabinan, and arabinogalactan bound with β-1,4 glycosidic bonds. Finally, the percentage of RG-II in plants is very low. However, it is extremely complex as it can have 12 different glycosidic residues (including glucuronic acid, ferulic acid, acetic acid, apiose, and fucose), which can be linked together with at least 22 different types of glycosidic bonds [94]. The latter plays an important role in the biomedical applications of Pec, exploiting the presence of carboxyl and hydroxyl groups that make it hydrophilic [95]. 

Chemically, Pec consists of poly α 1-4-galacturonic acids, with a variable degree of methylation of carboxylic acid residues [97]. According to the percentage or degree of methyl-esterification (DM) of α-D-galacturonic acid, Pec can be divided into Pec with a high methyl ester content (>50%, HM) and Pec with a low methyl ester content (<50%, LM) [60,61,98]. From a structural point of view, Pec has three different regions: a smooth region, defined as linear, a “hairy” region, and a branched region [99]. In fact, this polysaccharide can have arabinose, xylose, and galactose side chains [61], and its degree of esterification is variable and depends on many factors, such as plant storage and processing conditions, isolation, purification and extraction, and the origin of the plant from which it is extracted [99]. The degree of esterification of the galacturonic acid residues of Pec is the most important parameter affecting the solubility of Pec and its gelling and film properties [60,93,99].

Low degree of methylation (LM) Pec forms gels in the presence of multivalent ions such as aluminum Al^3+^ and calcium Ca^2+^, while HM Pec can gel in acidic solutions supplemented with different types of sugars, e.g., sucrose or glucose. In this case, gelation is induced by hydrogen bonds and hydrophobic interactions [57,93,98]. 

Due to its healthy, gelling, thickening, and emulsifying properties, Pec has been widely used for several decades in food and pharmaceutical fields [100,101]. Its application in the medical and drug delivery fields exploits its chemical nature and the presence of functional groups such as hydroxyl, amide, methyl, and carboxylic groups. In particular, the carboxylic group (-COOH) can be present in different forms in the molecule, such as -COO-, -COOH, and -COOH_2_^+^ [100,102,103]. 

Pec has the ability to regulate the pH of a solution, and in the presence of positive charges, such as Ch, gelatin, and casein, electrostatic interactions are generated [22]. In more detail, the -COOH functional groups can form a covalent bond with hydroxyl and amine groups [104,105]. In addition, electron-rich groups, including the hydroxyl group and the free carboxylic group, can interact with polyvalent metal ions such as Ca^2+^, Zn^2+^, and Fe^3+^ [100,106,107]. All these processes and chemical reactions lead to the formation of hydrogels, films, and microspheres [22,100,106,108]. In contrast to other polymers, such as cellulose, Ch, sodium alginate, and amide, Pec exhibits adverse effects on mechanical strength and chemical reactivity. Therefore, in biomedical applications, modified pectins are often used, as pure Pec often shows undesirable effects such as swelling and fast degradation [22]. To a lesser extent than Ch, Pec has been used for the development of three-dimensional culture systems. RGD-functionalized Pec hydrogel, obtained by internal gelation, supported in vitro culture of hMSCs [107]. Pectin gels crosslinked with CaCO_3_ have been successfully optimized as bioink and the possibility of exploiting such inks for 3D-printed brain models was studied by evaluating cell viability over time [109].

## 4. Ch and Pec Hybrid Hydrogel Systems

Since organs and tissues in vivo are extremely complex, often a single type of hydrogel does not satisfy entirely the target 3D cell culture requirements and, for this reason, the development of hybrid hydrogels represents a fruitful option [4]. In order to improve the performance of single polymer hydrogels, hybrid hydrogels, in which two or more biopolymers interact synergistically, can be developed through chemical and physical modifications and by co-polymerization [4,57,110]. These composite hydrogels exhibit more controlled physicochemical properties and are more representative of the remarkable complexity and dynamicity of the organ and tissue in vivo microenvironment [4]. These composites can be much more similar to the ECM of the native tissue than single component hydrogels, as they combine the properties of two or more materials [111,112], and the incorporation of a second material into the starting hydrogel allows the development of dual network (DN) hydrogels or interpenetrating polymer network (IPN) hydrogels, optimal for the representation of complex cell–matrix interactions and for obtaining improved mechanical properties [113]. 

IPN systems consist of the combination of independent but interdigitating polymer networks at the molecular level. These IPN systems, unlike polymer blends, exhibit better mechanical strength and drug loading ability, and more controlled swelling properties. Moreover, unlike composite hydrogels, if no cross-linkage breaking occurs, in IPN systems the polymer networks cannot be separated from each other [113]. Semi-IPN systems, on the other hand, can be obtained in a similar way to IPN systems. However, unlike IPN systems, they have lower mechanical properties and are obtained when incorporation of a second polymer occurs within a cross-linked hydrogel. The addition of a second polymer can occur by diffusion into an already cross-linked network or by selective cross-linking of a primary polymer with itself in the presence of a second polymer which is not cross-linked to the primary network [113]. Unlike IPN systems, semi-IPNs resemble polymer blends, as the secondary polymer can be “removed” from the primary network, without involving breakage of any chemical bonds [113]. Semi-IPN systems have been widely used for the controlled delivery of drugs, recombinant proteins, and growth factors [17,114,115].

**Figure 4:**
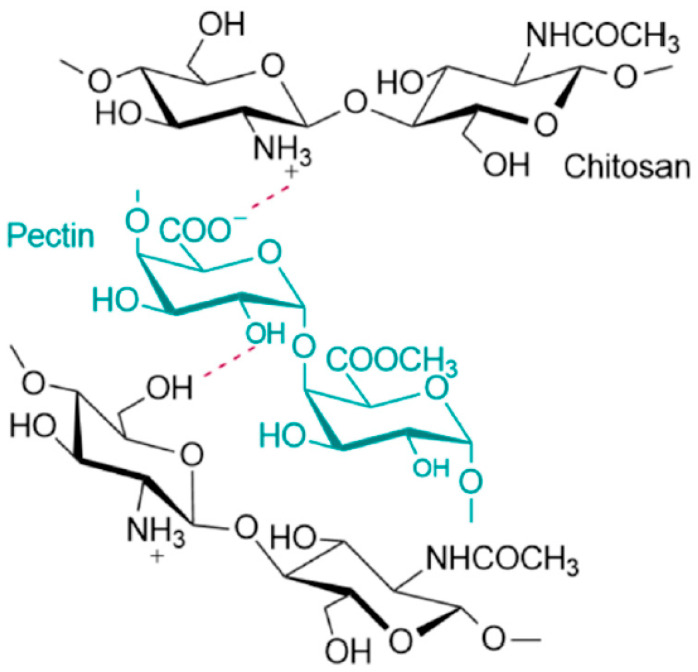
Representation of the intermolecular forces among Ch and Pec polymers [116].

Among hybrid hydrogels composed of natural polymers, Ch–Pec systems have recently been proposed for tissue engineering and drug delivery applications [59,116]. The interaction between the two polymers leads to the formation of a physical hydrogel with the development of an interpolymer complex network [60]. The complex occurs in an aqueous environment under certain pH conditions as a result of the electrostatic interactions between negative carboxyl groups of Pec and positive amino groups of Ch, as shown in Figure 4 [117]. 

In recent decades, Ch–Pec composite materials have been widely studied for their ease of use [95]. In the Pec molecule, the carboxyl group (-COOH) can dissociate into -COO-, generating electrostatic interactions with oppositely charged molecules when the pH value of the medium is higher than the pKa value of Pec, which is 3.6 [103,118]. If the pH value rises further, the ammonium molecules in Ch lose their protons, becoming amine groups (-NH_2_), and the electrostatic interaction disappears. On the contrary, when the solution has a pH value below 3.6 (Pec pKa), the carboxyl groups (-COOH) of Pec become neutral and the amino groups (-NH_2_) of Ch turn into (-NH^3+^), and electrostatic interaction does not occur [103,119,120,121].

## 5. Biomedical Applications of Ch–Pec Hydrogels

Several studies have shown that Ch–Pec composite hydrogels can be easily prepared by mixing Ch and Pec solutions in a pH range of 3 to 6 (acidic pH), leading to the formation of polyelectrolyte complexes between the positively charged Ch (polycation) and the negatively charged Pec (polyanion) [60,61]. However, an intensive study of the state of the art Ch–Pec composite systems revealed that the solubilization of the two polymer powders usually occurs in acidic solutions and at temperatures of 60 °C to 97 °C, which is not suitable for application as an injectable biomaterial (e.g., as an implantable hydrogel or bioink). Generally, the proposed hydrogels are in the gel state at room temperature (r.t.) and in the liquid state, therefore injectable, at high temperature [56,57,59,61,103,122,123,124,125,126].

In detail, the first work regarding the synergistic interaction between Ch and Pec was in 2003, when Norby and his research team studied the solubilization of the polymers in an acidic solution of 0.1 M hydrochloric acid (HCl) overnight at a temperature of 55 °C [60]. A similar process was adopted by Hiorth et al. in 2005, involving the mixing of polymers at a higher temperature (70 °C) for 1 h [57]. Similarly, Ventura’s team in 2015 dissolved Ch powder in 0.1 M HCl overnight at about 60 °C, and the same procedure was used for Pec powder; their mix was, finally, brought to 60 °C [61]. In 2011, Ch–Pec scaffolds for tissue engineering have been prepared by freeze-drying polyelectrolyte complexes. Ch–Pec scaffolds supported the adhesion and differentiation of human osteoblast cells, showing a good biocompatibility, as confirmed by MTT test results [127]. In the work by Birch and co-workers in 2015, the synthesis of a hydrogel under acidic conditions was addressed through dissolving Ch in acetic acid (CH_3_COOH) and Pec powder in deionized water (DI) at r.t. However, high solubilization temperatures of around 97 °C were needed [56]. Ch–Pec hydrogels were tested with human marrow-derived stem cells; protein absorption and cell proliferation were improved by rinsing the hydrogels. However, the low cell adhesion over 14 days of culture suggests that the hydrogels proposed by Birch and colleagues likely will not stick to wounds, thus facilitating the changing of dressings and therefore being potentially suitable for wound healing.

Conversely, Martins and colleagues [125] showed for the first time that a Ch–Pec membrane can promote attachment, adhesion, and proliferation of adipose-derived stem cells (ADSCs). In particular they demonstrated that by adjusting the Pec membrane composition, the hydrophilicity and hydrophobicity can be easily tuned, enabling interactions between ADSCs and the membrane surface.

**Figure 5:**
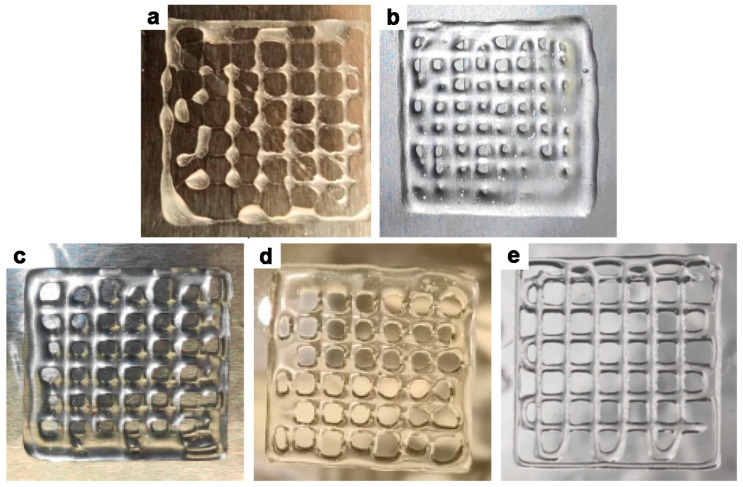
Photos of 3D printed (**a**) Ch solution 4%, (**b**) Ch-Pec 4–5%, (**c**) Ch-Pec 4–10%, (**d**) Ch-Pec 5–5%, and (**e**) Ch-Pec 5–10% *w/v* [117].

Ch and Pec solutions have also been combined to obtain cryogels [128]. Cryogels were characterized in vitro in terms of mechanical strength, swelling ability, surface morphology, adhesion, degradation, and biocompatibility. The results showed that the developed composite cryogels were biocompatible and could potentially be used in biomedicine and biotechnology. In the 2017 work of Tentor and coworkers, a Ch–Pec scaffold containing gold (Au) nanoparticles was developed for bone regeneration. The Ch powder (pw) was dissolved in 0.1 M HCl under stirring for 1 h at elevated temperatures (65 °C), and the same procedure was used for the Pec powder. Finally, to achieve gelation, the mix was kept at r.t. [122]. The authors tested the biocompatibility of Ch–Pec hydrogels with and without Au nanoparticles and reported that the presence of nanoparticles increased human colon adenocarcinoma cell (HT-29) viability from 67% to more than 90%. In the same time frame, research by Neufeld and collaborators in 2017 led to the design of Ch–Pec hydrogel vesicles for drug delivery. Specifically, the Ch powder was solubilized in 0.1 M HCl under stirring for 24 h at elevated temperatures (about 60 °C). The same procedure was implemented for the Pec powder, and the final mix of Ch and Pec was then stirred for 1 h at 60 °C [59]. Ch and Pec were also proposed as composite beads for antimicrobial applications, where the ionotropic gelation of Pec with CaCl_2_ was exploited. It was then dissolved in deionized water at 40 °C and combined with the salt solution and the solubilized Ch again in acetic acid at a temperature of 70 °C [123]. Towards the development of anatomically relevant structures as scaffolds for biomedical applications by 3D printing [116], or more specifically for wound healing [124], printable hydrogels of Ch–Pec have been developed and successfully 3D printed. Ch–Pec inks were prepared by powder solubilization in acetic acid, reaching the temperature of 80 °C to promote Pec solvation and the sol–gel transition at 50 °C, although cell colonization and growth was carried out on the pre-printed structures (Figure 5) [116].

Exploiting the hydrogel’s ability to be delivered in the tissue defect area, Ch–Pec have been proposed for the preparation of injectable hydrogels for articular cartilage treatment and regeneration [129]. With the aim of improving the mechanical properties of Ch–Pec hydrogels, cellulose nanofibers have been introduced as nanofillers. The injectable reinforced hydrogel can be prepared from Ch and Pec solutions in a liquid form by using a double-barrel syringe, which represents a useful strategy to increase the potential of Ch- and Pec-based hydrogels in the field of tissue engineering.

**Figure 6:**
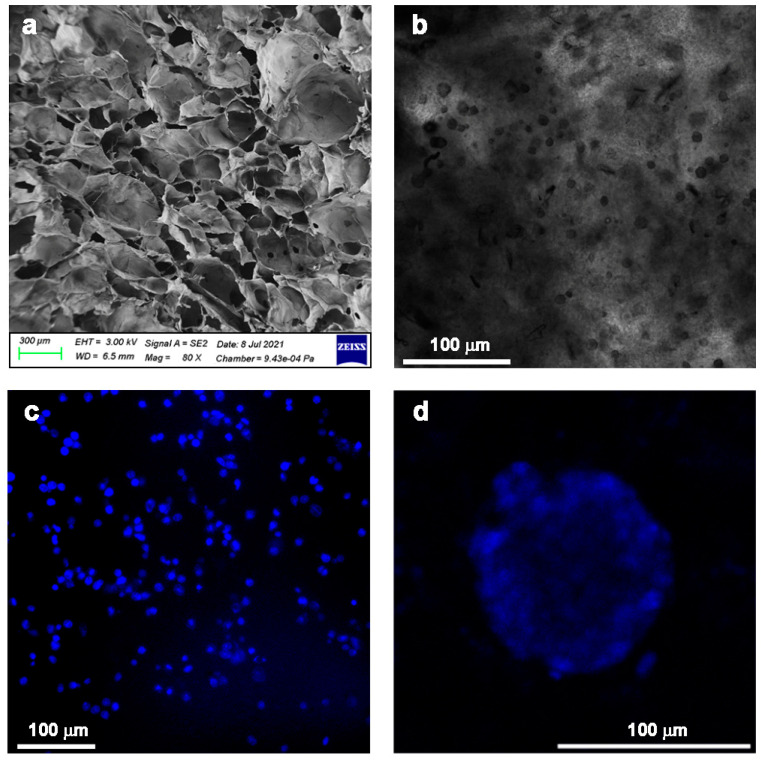
Injectable thermosensitive Ch–Pec IPN for colon rectal cancer in vitro modeling: (**a**) morphological analysis of the hydrogel structure by scanning electron microscopy; (**b**,**c**) bright field and fluorescence optical microscopy investigation at 24 h of HTC 116 cell culture in the Ch–Pec hydrogel; (**d**) fluorescence optical microscopy investigation of spheroids formed and grown in Ch–Pec hydrogels after 21 days of culture [131].

Finally, few studies have explored the possibility of preparing Ch–Pec hydrogel formulations in mild conditions, which would therefore be suitable for cell encapsulation. This would expand the potential use of these materials to many tissue engineering and in vitro modeling applications. Recently, an injectable thermosensitive Ch–Pec IPN was produced for embedding and culturing a colon rectal cancer cell line (HCT 116), without the need for strong acid conditions or high temperatures (Figure 6) [130]. The system was further investigated as a tumor matrix analogue and allowed the formation and long-term culture of HTC 116 spheroids, showing a direct relationship between the hydrogel composition, in terms of Ch–Pec concentration, and spheroid growth [43].

## 6. Conclusions

The development of physiologically relevant 3D tissue analogues has important outcomes for the fields of tissue and organ regeneration and in vitro modeling. The urgent need for successful substitutes to repair damaged tissues is well recognized by the clinical community. In addition, the search for new methodologies to study and understand the mechanisms that regulate organ physiology or pathogenesis involves the use of 3D in vitro models that can overcome the limitations of current 2D culture techniques and animal models. Indeed, in recent years, increasing attention has been paid to the development of sophisticated 3D in vitro tissue models for studying the pathophysiological mechanisms underlying organ function and disease [131,132,133]. 

Hydrogels, acting as 3D supporting architectures and allowing cells to spatially organize themselves in a manner more similar to what they experience physiologically in vivo, represent a key element in the development of 3D tissue analogues. In this scenario, hydrogels based on natural polymers, such as Ch and Pec, two versatile polysaccharides, are an essential tool for tissue engineering and 3D in vitro modeling. 

Given the great complexity of organs and tissues in vivo, hydrogels made of a single polymer do not always satisfy the full range of requirements. Hybrid hydrogels, made of two or more polymers, are a fruitful option. Ch and Pec, in particular, are very versatile carbohydrates that can be easily processed and combined to finely tune the hydrogel stiffness and increase the usually short stability of a single natural polymer-based hydrogel. The possibility of accurately modulating the physico-chemical properties of a cell-embedding hydrogel has important outcomes on the quality of tissue regenerated by cells in vitro, as the regeneration processes, as well known, are strongly influenced by mechanical factors. However, the natural origin of the two starting polymers could represent a drawback in terms of batch-to-batch variability and, as a consequence, repeatability issues may arise. 

This literature review on Ch and Pec hydrogel systems has shown how they are able to cover the broad spectrum of properties required for an ideal hydrogel in terms of strength and degradation with respect to tissue engineering and 3D in vitro modeling applications.

Currently, the manufacturing processes and standardization of Ch and Pec hydrogels still need to be investigated and improved to implement their use in the medical field. However, hydrogels based on natural polymers such as Ch and Pec, which are widely available and low-cost biopolymers, will increasingly find widespread use in specific medical applications due to advances in the field.

## Data Availability

Data are contained within the article.
